# Improving interMediAte Risk management. MARK study

**DOI:** 10.1186/1471-2261-11-61

**Published:** 2011-10-13

**Authors:** Ruth Martí, Dídac Parramon, Luís García-Ortiz, Fernando Rigo, Manuel A Gómez-Marcos, Irene Sempere, Natividad García-Regalado, Jose I Recio-Rodriguez, Cristina Agudo-Conde, Natalia Feuerbach, Maria Garcia-Gil, Anna Ponjoan, Miquel Quesada, Rafel Ramos

**Affiliations:** 1 Unitat d'Investigació en Atenció Primària de Girona, IDIAP Jordi Gol, Institut Català de la Salut, C/Maluquer Salvador, 11, Girona, 17071, Espanya; 2 Institut d'Investigació Biomèdica de Girona Dr. Josep Trueta (IDIBGI), Av. de França s/n, Girona, 17007, Espanya; 3 Unidad de Investigación de Atención Primaria La Alamedilla, Centro de Salud La Alamedilla, Av Comuneros 27-31, Salamanca, 37003, España; 4 CS San Agustín, Gerencia Atención Primaria Ibsalut, C/Nicolau Alemany 1, Palma de Mallorca, 07015, España; 5 Departament de Ciències Mèdiques. Facultat de Medicina Universitat de Girona, Campus de Montilivi, Girona, 17071, Espanya

**Keywords:** Risk assessment, cardiovascular diseases, primary prevention, primary health care.

## Abstract

**Background:**

Cardiovascular risk functions fail to identify more than 50% of patients who develop cardiovascular disease. This is especially evident in the intermediate-risk patients in which clinical management becomes difficult. Our purpose is to analyze if ankle-brachial index (ABI), measures of arterial stiffness, postprandial glucose, glycosylated hemoglobin, self-measured blood pressure and presence of comorbidity are independently associated to incidence of vascular events and whether they can improve the predictive capacity of current risk equations in the intermediate-risk population.

**Methods/Design:**

This project involves 3 groups belonging to REDIAPP (RETICS RD06/0018) from 3 Spanish regions. We will recruit a multicenter cohort of 2688 patients at intermediate risk (coronary risk between 5 and 15% or vascular death risk between 3-5% over 10 years) and no history of atherosclerotic disease, selected at random. We will record socio-demographic data, information on diet, physical activity, comorbidity and intermittent claudication. We will measure ABI, pulse wave velocity and cardio ankle vascular index at rest and after a light intensity exercise. Blood pressure and anthropometric data will be also recorded. We will also quantify lipids, glucose and glycosylated hemoglobin in a fasting blood sample and postprandial capillary glucose. Eighteen months after the recruitment, patients will be followed up to determine the incidence of vascular events (later follow-ups are planned at 5 and 10 years). We will analyze whether the new proposed risk factors contribute to improve the risk functions based on classic risk factors.

**Discussion:**

Primary prevention of cardiovascular diseases is a priority in public health policy of developed and developing countries. The fundamental strategy consists in identifying people in a high risk situation in which preventive measures are effective and efficient. Improvement of these predictions in our country will have an immediate, clinical and welfare impact and a short term public health effect.

**Trial Registration:**

Clinical Trials.gov Identifier: NCT01428934

## Background

In Spain, as in most Mediterranean countries, death rates from cardiovascular diseases have traditionally been lower compared with those in Anglo-Saxon and northern and central Europe [1]. However, they are still a priority public health problem because vascular diseases contribute to 30% of total mortality in developed countries, 56 million deaths worldwide, of which more than 125, 000 are in Spain [2]. The European guideline for vascular prevention [3] recommends that prevention strategies should include three main components: 1) a population strategy to try to change lifestyle, social, environmental and economic factors which are highly related to the disease occurrence 2) a secondary prevention strategy to reduce recurrences, complications rates and improve the disease prognostic and 3) a primary prevention strategy in high-risk population in which different interventions are more efficient than in general and low-risk population. In this last strategy, the key element is to identify these individuals through the use of risk functions. This strategy has some limitations at present, since sometimes the performance of such functions is insufficient, especially in patients classified as intermediate-risk (5-15% risk at 10 years) [4]. Several attempts to improve risk functions by using new risk factors markers have not increased their predictive ability, because of the autocorrelation of these emerging factors with classical risk ones [5,6]. With the available evidence we cannot assess the balance between risks and benefits of the studied factors for its widespread use in asymptomatic men and women with no history of coronary disease [7]. The general conclusion is that this issue is a priority in vascular prevention research.

There are three opportunity areas to improve the risk estimation in intermediate-risk population:

1. The measure of subclinical atherosclerosis: Markers of atherosclerotic burden are the most reliable candidates to identify patients at increased risk of the intermediate-risk group [8]. Values below 0.9 on the ankle brachial index (ABI) are associated with an increased risk of coronary heart disease events and mortality, regardless of calculated risk by the Framingham equation [9], even in asymptomatic patients [10]. Therefore, ABI at baseline provides extra prognostic information which is not provided by the measurement of conventional risk factors alone. In our country, results have also been reported to confirm this assumption, since ABI values < 0.9 have been found to be relatively common in asymptomatic patients with coronary risk at 10 years less than 10% [11].

Arterial stiffness is also a parameter that is considered as a possible good marker of cardiovascular risk [12,13]. Traditionally, it has been quantified by pulse wave velocity (PWV). The Cardio Ankle Vascular Index (CAVI) is an index representing the stiffness of the aorta, femoral artery and tibial artery [14]. Some authors suggest that CAVI measurement, which is independent of blood pressure and has an adequate reproducibility for clinical use, is more useful as a marker of arterial stiffness than PWV [15].

2. The incorporation of more detailed and accurate assessment of traditional risk factors: The glycated hemoglobin (HbA1c) is a more accurate and stable measure than fasting glucose concentration [16]. It is more and more suggested that HbA1c concentration is not only a useful marker in patients with DM, but can also be useful to identify patients at higher risk of developing cardiovascular problems in the general population. In this regard, a recent study shows that high levels of HbA1c are associated with the occurrence of cardiovascular disease in nondiabetic patients [17] and high values of this type of hemoglobin are common in general adult population with no history of diabetes [18].

Postprandial glucose concentration has negative effects on the arterial wall and several studies have shown that a good control of postprandial glucose can revert to a reduction of cardiovascular events and mortality [19,20].

Self-measured blood pressure has a significant correlation with cardiovascular morbidity and mortality [21] and its control has some advantages such as being easily accessible and comfortable for the patient. It also has the possibility of performing multiple measurements on non-medical environment, so that white coat effect is avoided.

3. The incorporation of a comprehensive and contextualized evaluation of the patient taking into account his co-morbidity: Several studies have highlighted the importance of the presence of certain chronic diseases in vascular diseases development. There are arguments supporting that the presence of chronic obstructive pulmonary disease [22], atrial fibrillation [23], renal disease [23] and depression [24] among others are associated, independently, with an increased incidence of vascular disease. Just as the high score on composite indices of co-morbidity, has been associated with a worse prognosis of vascular diseases [25]. This suggests that this information may be useful for risk stratification, even in primary prevention.

Thus, these three lines of analysis and the proposed variables have significant scientific support as candidates to improve the population risk classification. They are also measures that can be easily and economically obtained and can be implemented in primary care, which is suitable for the population screening. The demonstration of the usefulness of some of these factors in re-stratify the population's risk will have a major impact on the primary prevention of the vascular diseases with all that this implies at the Public Health level. All this justifies the proposal to analyze their contribution to improve the predictive ability of the existing risk functions.

### Objectives

To analyze whether the ABI and arterial stiffness measures (pulse wave velocity and cardio ankle vascular index) are associated independently with the incidence of vascular events and whether they can help to improve the predictive ability of risk equations based on traditional risk factors in intermediate-risk population.

To analyze whether the postprandial capillary glucose, glycated hemoglobin, and self-blood pressure measurement are associated independently with the incidence of vascular events and whether they can help to improve the predictive ability of risk equations based on classical risk factors in intermediate-risk population.

To analyze whether the presence of comorbidity measured by composite indices (Charlson Index, Cumulative Illness Rating score) is associated independently with the incidence of vascular events and whether they can help to improve the predictive ability of risk equations based on classical risk factors in intermediate-risk population.

## Methods/Design

### Design

Multicentre prospective cohort study with a follow-up to 1.5, 5, and 10 years from the participant inclusion in the study.

### Subjects

#### Study population

Population aged between 35 to 74 years who have an intermediate cardiovascular risk, defined as coronary risk between 5% -15% at 10 years according to the Framingham adapted risk equation (because of the low incidence of coronary heart disease in our country) [26] or vascular mortality risk between 3-5% at 10 years according to the SCORE equation [27]. Exclusion criteria: terminal illness or institutionalization at the appointment time or a personal history of atherosclerotic disease. Sample selection: There will be a random sample of population aged 35 to 74 (both included) which have an intermediate cardiovascular risk. This is a multicenter project in the context of the Instituto Carlos III Preventive and Health Promotion Research Network (RedIAPP) (RETICS RD06/0018). Sample recruitment will be carried out in Health Centers belonging to 3 groups of the 3 network regions. Candidate patients will be cited by telephone at their health centre and invited to participate by signing an informed consent.

#### Sample size

The ABI < 0.9 population proportion with no history of vascular disease has been taken into account because ABI is the risk factor among those studied that we anticipate will have a lower prevalence. It is expected that in one year and a half of follow up 3% of the participants will present a vascular event. Therefore, accepting a 0.05 alpha risk and a 0.2 beta risk in a bilateral contrast, 336 subjects are needed in the exposed group and 2352 in the non-exposed group (2688 total) to detect a minimum relative risk of 2. We assumed an incidence rate in the unexposed group of 3%. Estimating a 25% of loss rate until 3360 participants will be oversampled. Poisson approximation has been used. The estimated relative risk is based on previous observational studies in which patients of the risk group had twice the incidence of vascular disease if they had an ABI < 0.9 [9]. In Catalonia node centers 1900 participants are expected to be recruited and 400 participants in each of the other two nodes. Professional nurses assigned to each health center will perform the physical examination and questionnaires. They will explain how to record the self-measurement of blood pressure and capillary blood glucose and the candidates will be cited later to collect this information. At the same time they will be scheduled for a fast blood test.

#### Data collection method

A case report form has been created for the study. For telephone follow-up a questionnaire has been created to record death or vascular events.

### Variables

It will be recorded the demographic baseline information of age, sex, marital status and employment status. Educational level and social class will also be recorded as potential confounding factors. Educational level will be grouped into three categories: high level (patients with university education), middle level (secondary education until age 16 -18) and low level (patients who haven't received any education or have only completed primary school). Social class will be measured by the Spanish National Occupation Classification. Drugs which may be related to cardiovascular diseases will be recorded (antiplatelet agents, warfarin, oral contraceptives, hormonal therapy, lipid lowering agents, antihypertensive and antidiabetic agents).

#### Co-morbidity study

Clinical history, date of diagnosis and treatment of hypertension, diabetes, atrial fibrillation, chronic obstructive pulmonary disease, renal disease and depression will be collected. The Charlson Comorbidity Index, which contains 19 comorbidity categories, will be calculated. Each index category has an associated weight, taken from the original Charlson's document [28], which is based on the adjusted mortality risk per year. The overall comorbidity score reflects the greater cumulative probability of mortality per year. The higher the score the more severe the comorbidity burden is. The Cumulative Illness Rating Scale (CIRS) will also be calculated. This index scores medical problems categorized into 14 organ systems from 0 to 4 points [29]. The general rules for the severity rating are the following: (0) no problem affecting this system or a problem of the past without clinical relevance, (1) current mild problem or severe problem of the past, (2) moderate disability or morbidity and/or requires a first-line therapy, (3) serious problem and/or significant disability and/or chronic conditions which are difficult to control (complex treatment regimen), (4) extremely serious problem and/or required immediate treatment and/or organ failure and/or severe functional impairment.

#### Diet

A validated food frequency consumption questionnaire [30] and a validated questionnaire about the adherence in Mediterranean diet [31] will be used.

#### Physical activity

The adapted Minnesota's leisure time activity questionnaire will be used, whose use has been validated in the Spanish population [32]. The average daily physical activity during leisure time of the last year will be reported.

#### Tobacco consumption

A questionnaire of 4 standard questions adapted from the WHO MONICA study will be used. The carbon monoxide concentration will be measured by a co-oximeter and the participants will be asked when the last cigarette was smoked.

#### Alcohol consumption

A questionnaire about alcohol consumption in the last 7 days will be included through a detailed questionnaire about alcohol types and volume.

#### Intermittent claudication

The Edinburgh validated questionnaire will be used [33]. Existence of asymptomatic PAD will be considered when ABI values are < 0.9 and no intermitent claudication are registered in the questionnaire.

#### Electrocardiogram

A 12-derivation digital electrocardiograph CardioSoft^® ^(General Electrics) will be used to test the participants at rest.

#### Blood pressure measurements

Blood pressure measured by nurse will be made in sitting position after 5 minutes of rest and it will be performed 3 times separated by 2 minutes in each arm. An automatic and validated device will be used (OMRON 705). The subject should avoid doing any type of exercise at least one hour before the measurements. He or she should also avoid eating or drinking much, smoking and taking medication that could directly affect the BP (except in hypertension treated patients). Blood pressure self-measurement will be made by participants at their home for three days. They will make 2 blood pressure measurements separated by 2 minutes in the morning and in the evening, before having breakfast and dinner, respectively [34].

#### Ankle-brachial index and arterial stiffness

They will be measured using Vasera device VS-1500^® ^(Fukuda Denshi). For the study, the lowest ankle-brachial index obtained will be considered. The pulse wave velocity (PWV) will be calculated, as well as Cardio Ankle Vascular Index (CAVI), which gives a more accurate calculation of the atherosclerosis degree. CAVI integrates cardiovascular elasticity derived from the aorta to the ankle pulse velocity through an oscillometric method and it is used as a good measure of vascular stiffness. It doesn't depend on blood pressure and the information that it gives may help to prevent peripheral vascular occlusions [14]. Ankle-brachial index, blood pressure and CAVI will be measured at rest and after light intensity exercise in order to evaluate whether there are changes in the measurements that can predict cardiovascular risk better. Participants will cycle on a static bike for 5 minutes at 70 wats of power before the measurement.

#### Anthropometric measurements

Weight will be measured with a precision scale, which will be weekly calibrated. The return to zero will be checked after every measurement. Size will be measured vertically. Subjects should remove their shoes, jackets, coats etc. Waist circumference: the midway between the lowest rib and the iliac crest will be localized.

#### Laboratory data

The blood sample will be taken after 12 hours fasting. Cholesterol and triglycerides concentration will be determined by enzymatic methods and HDL cholesterol after apo B containing lipoprotein precipitation. LDL cholesterol will be determined by the Friedewald formula. Glucose concentration (mg/dl), glycated hemoglobin (%) and creatinine will also be determined, as well as urine albumin, to calculate the albumin/creatinine index. Postprandial glucose (mg/dl) will be self-measured by patients at home 2 hours after meals (breakfast, lunch and dinner) for one day using an Accu-chek ^® ^glucometer.

#### Vascular events in the follow-up

There will be a telephone follow-up to the 1.5, 5 and 10 years to verify the vital status and the existence of hospital admissions due to vascular health problems. The information of each case will be confirmed after a review of hospital discharge reports and the verification of the diagnostic criteria by a consensus of at least 2 investigators. Fatal and non fatal coronary heart disease (myocardial infarction or angina pectoris) will be considered. Cases will be classified according to symptoms, electrocardiogram, and myocardial necrosis markers. Fatal cases will be classified through the death certificate or autopsy findings. Angina pectoris only will be considered if there are changes in the ECG or a positive stress test. Stroke will be considered when neurologic deficit lasting more than 24 h attributable to a diagnosis of a focal cerebral ischemia is confirmed by an imaging test (CT or MRI). Peripheral arterial disease will be considered if there is a diagnostic made by arteriography or Doppler (ankle-brachial index below 0.9) or lower limb amputation or ulcer or gangrene attributable to an ischemic deficit.

### Statistical analysis

Percentages will be used to describe categorical variables. Continuous variables will be described with the mean and standard deviation or with the median and the interquartile range as appropriate. Homogeneity among centers will be proved by a Chi-square test or Kruskal-Wallis test depending on the probability distribution of variables. A multivariate analysis by Cox proportional risk models will be performed to examine the contribution of the studied risk factors adjusted by traditional risk factors (age, sex, total cholesterol and HDL cholesterol, smoking status, diabetes and blood pressure).

### Limitations and biases of the study

One of the major difficulties of the study is to achieve a good participation rate. The research team has experience in cohort studies with good success rates (above 70%). Some of the measurements which must be performed are laborious and require a previous training of people who will perform it to ensure accurate, comparable and quality results. This will be fixed by the previous training and a pilot test of 100 participants.

### Ethical considerations

The study has received ethical approval from the Research Ethics Committee of the Institut d'Investigació en Atenció Primària Jordi Gol (Primary Care Research Institute Jordi Gol). Participants will be informed about the examinations performed, they will receive an information document and they will be asked for the informed consent prior to their inclusion. The principles of human experimentation, as the Helsinki Agreement, will be respected. Access to the information obtained from any analysis will be guaranteed. Confidentiality rules will be respected and the participants will be informed about the 5^th ^Article of the15/1999 Spanish organic Law, about the regulation of the automatic processing of personal data (personal data may be automatically processed and the participants have the rights to consult, modify or delete their personal data file).

## Discussion

Primary prevention of cardiovascular diseases is a priority in public health policy of developed and developing countries. The fundamental strategy consists in identifying people in a high risk situation in which preventive measures are effective and efficient. However, specificity and sensitivity of risk equations are modest, which means that approximately 50% of the patients who are likely to develop a vascular event and would benefit from preventive measures are not considered at high risk. While 30% of the subjects considered at risk don't really benefit from preventive measures. Moreover, decisions which imply thousands of people and can determine drug treatment indications are taken every day in primary care centers. These decisions are based mostly on the result of estimations about the probability to develop a vascular disease in 10 years. Improvement of these predictions in our country will have an immediate, clinical and welfare impact and a short term public health effect. The efficiency and therefore the best use of the resources will benefit our public health system immediately. The global relevance of the issue about improving predictive risk models, the novelty of including determinate risk factors in a southern Europe population and to build equations for risk subgroups amply justifies the implementation of this project.

## Competing interests

The authors declare that they have no competing interests.

## Authors' contributions

Conception of the idea for the study: RR, RM and MG. Development of the protocol, organization and funding: RR, RM, DP, LGO, FR, MAG, IS, NGR, JIR, CAC, NF, MG, MQ and AP. Writing of the manuscript: RR, RM, MG and DP. All the authors have read the draft critically, to make contributions, and have read and approved the final manuscript. The project will be developed by cardiovascular REDIAPP (Preventive Services and Health Promotion Research Network).

## Pre-publication history

The pre-publication history for this paper can be accessed here:

http://www.biomedcentral.com/1471-2261/11/61/prepub
